# Nucleic acid-lipid membrane interactions studied by DSC

**DOI:** 10.4103/0975-7406.76470

**Published:** 2011

**Authors:** Sarantis Giatrellis, George Nounesis

**Affiliations:** Department of Medical Biochemistry and Biophysics, Umeå University, SE-901 87 Umeå, Sweden; 1 Biomolecular Physics Laboratory, IRRP, National Centre for Scientific Research “Demokritos”, 153 10 Aghia Paraskevi, Greece

**Keywords:** Differential scanning calorimetry, DNA, DSC, lipid membranes, lipoplexes, liposomes, nucleic acids, RNA

## Abstract

The interactions of nucleic acids with lipid membranes are of great importance for biological mechanisms as well as for biotechnological applications in gene delivery and drug carriers. The optimization of liposomal vectors for clinical use is absolutely dependent upon the formation mechanisms, the morphology, and the molecular organization of the lipoplexes, that is, the complexes of lipid membranes with DNA. Differential scanning calorimetry (DSC) has emerged as an efficient and relatively easy-to-operate experimental technique that can straightforwardly provide data related to the thermodynamics and the kinetics of the DNA—lipid complexation and especially to the lipid organization and phase transitions within the membrane. In this review, we summarize DSC studies considering nucleic acid—membrane systems, accentuating DSC capabilities, and data analysis. Published work involving cationic, anionic, and zwitterionic lipids as well as lipid mixtures interacting with RNA and DNA of different sizes and conformations are included. It is shown that despite limitations, issues such as DNA- or RNA-induced phase separation and microdomain lipid segregation, liposomal aggregation and fusion, alterations of the lipid long-range molecular order, as well as membrane-induced structural changes of the nucleic acids can be efficiently treated by systematic high-sensitivity DSC studies.

Important biological mechanisms like chromatin function, as well as biomedical applications involving liposomal carriers for DNA delivery for clinical use in gene therapy, are based upon the incorporation of nucleic acids within lipid membranes.[1–4] Lipoplexes, that is, complexes of lipids with DNA that have been successfully used as transfection agents for gene delivery applications and viral capsids, where DNA– or RNA–lipid interactions take place, are two of the numerous examples manifesting the great importance of unraveling the detailed mechanisms of lipid–nucleic acid complexation.[5–9] Understanding the phases and the molecular organization of lipid membranes and the structures of their complexes with nucleic acids is essential for optimizing gene delivery efficiency and understanding the biological mechanisms at the molecular level.

The enormous possibilities presented by liposomes, exhibiting versatility with respect to their size, fluidity, and surface charge, have over the last 20 years paved the way for systematic research in the direction of developing stable liposomal complexes with proteins, peptides, metabolites, and drugs.[10] A large amount of work has already been published presenting results from extensive studies of interactions of lipid membranes with biomolecules, primarily explored by DSC, where the molecular order of lipids and the thermally induced phase transitions were probed.[11–13] Even though DSC proved to be a powerful and easy-to-operate technique, it became clear that for the correct interpretation and the deeper understanding of the calorimetric data, that is, heat capacity vs. temperature, the correlation of DSC with structural information from a variety of combined techniques was necessary.[14–19]

The negative charge density of the DNA molecule makes the direct electrostatic interaction with cationic liposomes possible. The addition of DNA to liposomes brings about substantial changes related to the molecular order and the structure of the lipid membrane. These changes heavily depend upon the fluidity of the membrane, the surface charge, and often the size of the preformed cationic liposomes. In this review, we summarize the nucleic acids–membrane interactions studied via DSC. These interactions depend upon parameters such as electrostatic interactions, lipid structure and membrane composition, entropic contributions, mesoscale conformations of membranes, and nucleic acids properties. DSC has definite limitations for studying thermal processes even more so for extracting detailed information about molecular order and structure. Despite these limitations, DSC is a sound technique for exploring membrane–biomolecules interactions through changes demonstrated in the calorimetric trace of lipid phase transitions. By employing thermodynamic analysis, high-sensitivity DSC has become a systematic method for investigating DNA or RNA interactions with lipid bilayers, shedding light into phase separation, lipid segregation, liposomal aggregation and fusion, as well as membrane-induced structural changes of the nucleic acids.

## Lipid Phases and Phase Transitions

Lipid molecules, when dispersed in aqueous media, self-organize spontaneously to a great variety of supramolecular structures depending upon the physicochemical properties of the lipids and the dispersant. This amazing polymorphism of lipids mainly includes lamellar, micellar, hexagonal, cubic normal, and inverted phases.[20,21] These phases and their transitions have been a broad field of research for many years because of their structural and functional biological importance.[22,23]

DSC is the experimental technique of choice to systematically characterize the thermodynamics of phase transitions and conformational changes of biological macromolecules and supramolecular structures. DSC measures isobaric changes in specific heat capacity as a function of temperature.[24–26] Thermal events like phase transitions absorb or release heat upon heating or cooling of the system, and thus information can be retrieved pertaining on the transition temperature, the enthalpy change, and the cooperativity of the transition as it can be inferred from the heat capacity peak with straightforward analysis. Single transitions, multiple transitions, phase separation, and aggregation phenomena can be distinguished. By comparing heat capacity curves and entailed molecular order changes – baring a cooperative character – we can extract qualitative and quantitative information about the physical chemistry of mechanisms and interactions on a molecular level.

DNA and RNA of a great variety in size and origin have been used for interaction studies (bovine/plasmid/herring/salmon 1–40 kbp). Synthetic lipid membranes have been tested either as multilamellar vesicles (MLVs) or as large unilamellar vesicles (LUVs). Lipid compositions assayed varied from pure zwitterionic phosphatidylcholine (PC) membranes to three lipid systems containing cationic compounds, fusogenic-helper lipids, or anionic lipids. Lipid phases involved were mainly lamellar (gel or liquid-crystalline) as well as inverted hexagonal or cubic.[10] DSC could reveal the effect of nucleic acids binding to the lipid bilayers through the changes induced upon the molecular order, and thus upon the phases and phase transitions.

## DNA–membrane Interactions

A systematic study of the complexation of cationic liposomes with DNA providing evidence that the lipid hydrophobic part plays a crucial role in the interaction with DNA was published by Subramanian *et al*.[27] The study straightforwardly demonstrated the lipid phase separation within the liposomal membranes as well as the lipid-induced abolishment of DNA thermal denaturing transition. More specifically, Subramanian *et al*. investigated the formation of lipoplexes from calf thymus DNA and binary lipid multilamellar liposomes composed of 1,2-dimyristoyl-sn-glycero-3-phosphocholine (DMPC) and one of the three synthetic cationic amphiphiles – bis[2-(11-phenoxyundecanoate)ethyl] dimethylammonium bromide (BPDAB), bis[2- (11-butyloxyundecanoate)ethyl] dimethylammonium bromide (BBDAB), and *N*-hexadecyl-*N*-{10-[*O*-(4-acetoxy)-phenylundecanoate] ethyl} dimethylammonium bromide (HADAB). All cationic lipids (CLs) bear a dimethylammonium bromide headgroup (DAB) as the DNA-binding moiety yet different hydrophobic parts.[27,28] Importantly, the miscibility of the CLs with DMPC was assessed by scanning liposomes composed of CL-DMPC mixtures with molar ratios from 0 to 1. The DSC profiles manifested that maximum miscibility was achieved for low CL to DMPC molar ratios (≤0.15). At intermediate and high molar ratios, multiple overlapping peaks were observed, proof of the formation of phase-separated domains. Particularly interesting phase diagrams for the three binary mixtures were constructed, based on the DSC data, demonstrating the existence of extended gel and fluid-phase coexistence regions. The DSC results demonstrated very different phase behavior depending on the lipid hydrophobic part.

A DASM-4 (Bioripor, Pushchino, Russia) calorimeter was employed for the study. The addition of even 0.2 mM calf thymus DNA to the cationic liposomes produced lipoplexes and induced dramatic effects in the DSC thermograms. Of course, the lack of interaction between the zwitterionic DMPC and DNA produced no changes in the DSC profiles of pure DMPC MLVs.[29,30] The hydrophobic part of the cationic components affected the degree of the DNA effect on the DSC thermograms. At low ratios, DNA-induced lipid demixing resulted to phase separation; a single cooperative thermal anomaly was split into two broader peaks in the presence of DNA. As DNA concentration increased, the main phase transition temperature was suppressed and the enthalpic content of the peak was decreased. An additional heat capacity peak emerged at a higher temperature than the main-phase transition attributed to CL-rich domains. Despite the main-phase transition enthalpy decrease, the total enthalpy of both transitions was larger than the initial. At higher nucleotide per CL ratio, the main-phase transition peak became broader, and the main-phase transition enthalpy increased, while the new peak had reduced enthalpy until complete abolishment at a molar ratio of 8. The DNA effect on vesicles composed of nonaromatic hydrophobic chains BBDAB was qualitatively the same as in the case of the diphenyl hydrophobic chains of BPDAB. In the case of the incompatible tail components, phenylamine in HADAB differentiated the effects enhancing the disorder induction by DNA even at low HADAB to DMPC ratio.

The zwitterionic lipid 1,2-dioleoylphosphatidylethanolamine (DOPE) has been widely used as a helper lipid in cationic lipid membranes for lipoplex formation as it enhances gene transfection efficiency due to its fusogenic influence. It is thus essential to study the properties of DOPE-containing membranes interacting with DNA.[31–38] Several DSC studies have been guided in this direction. Barreleiro *et al*. used dioctadecyl-dimethylammonium bromide - 1,2-dioleoyl-phosphatidylethanolamine (DODAB-DOPE) liposomes scanned by DSC to show that the inclusion of DOPE increased the fluidity of the membranes leading to phase separation at high DOPE contents.[39] The addition of DNA resulted in increased cooperativity for the main lipid phase transition, in case of pure DODAB, but it enhanced phase separation in the DODAB-DOPE dispersions. Saunders *et al*. have studied the effect of DOPE in ethyldimyristoyl-*O*-phosphatidylcholine (EDMPC) LUVs at various ratios and subsequently the effect of plasmid DNA (9 kbp) binding, by employing high-sensitivity DSC.[40] EDMPC-DOPE liposomes showed increased transfection efficiency as found by Gorman *et al*.[41] EDMPC is derived from DMPC by placing an ethyl to one of the phosphate oxygens resulting in a cationic lipid from a zwitterionic. Pure EDMPC vesicles exhibit a relatively cooperative main-phase transition with a low transition temperature (*T*_m_) shoulder attributed to an interdigitated phase. As in the previously described case of DODAB-DOPE, the addition of DOPE at 12% severely alters the lipid organization in the membrane by inducing higher membrane fluidity. It suppresses the main-phase transition peak while abolishing lipid interdigitation. Interestingly, a low-enthalpy DSC peak at a higher temperature than the main-phase transition provides proof of pure EDMPC domains in the mixture.

The addition of plasmid DNA, at a DNA-to-EDMPC charge ratio of 0.5, reduces the enthalpy by ~7%, while it decreases drastically (110%) the half width at half maximum of the heat capacity peak indicating an increase in cooperativity. As DNA binds electrostaticaly to EDMPC headgroup, long-range order may be enhanced leading to the observed increase in the cooperativity of the main-phase transition. On the other hand, the more fluid EDMPC-DOPE membranes exhibit more dramatic effects when bound to DNA. The main-phase transition peak is suppressed in both temperature and enthalpy, in favor of the EDMPC-rich phase-separated regions. Interestingly, for EDMPC-DOPE liposomes at 50% mol DOPE, while noncomplexed liposomes present no DSC features, the DNA-complexed ones exhibit two distinct low-enthalpy peaks characteristic of the structural changes induced by the binding of DNA.

**Figure 1:**
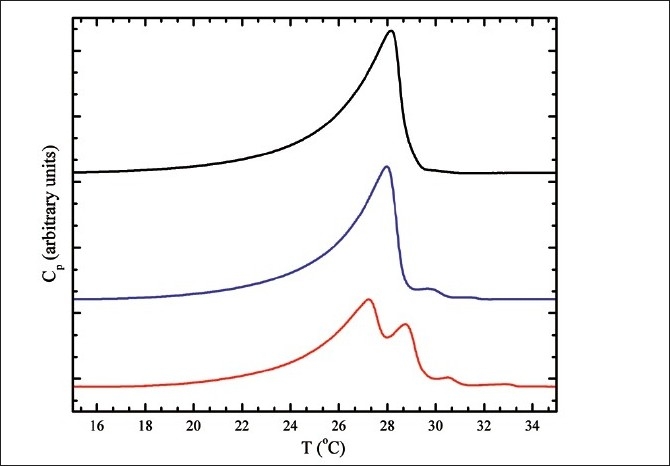
Characteristic DSC traces for DMTAP-DOTAP 4:1 multilamellar vesicles (top curve), DMTAP-DOTAP 4:1 – plasmid DNA complex first heating scan (middle curve) and second scan (bottom curve). Experimental procedures are described in Giatrellis *et al*.[43]

When DNA is incorporated within the lipid membranes, the changes that occur are quite dramatic, as described before. The interactions are also very slow and in order to characterize them properly several consecutive heating and cooling DSC scans are required. This was particularly pronounced in a case of DNA-mediated segregation of lipid binary mixtures that differ in their aliphatic chains, which was observed in a study by Giatrellis *et al*., of binary 1,2-dimyristoyl-trimethylammonium propane - 1,2-dioleoyl-trimethylammonium propane (DMTAP-DOTAP) lipoplexes.[42] Upon consecutive heating scans, the single thermal anomaly from the neat DMTAP-DOTAP unilamellar liposomes main-phase transition that was originally detected underwent changes in the transition temperature and also in the number of DSC peaks and their total enthalpy. During the very first cycle from gel to liquid-crystalline phase and back to gel phase, multiple DSC peaks emerged; the highest temperature peak coincides with the temperature of the phase transition of multilamellar DMTAP-DOTAP vesicles. This can have multiple interpretations; DNA interconnects and restricts trimethylammonium propane (TAP) headgroups, while at the same time DNA can induce multilamellarity, and finally DNA might be selective to the more fluid DOTAP lipids and thus creates DMTAP-rich domains.[43,44] All three scenarios can be concurrent, as they are also supported by light-scattering particle size measurements as well as by isothermal titration calorimetry experiments. Following a number of successive order–disorder bilayer transitions, the abolishment of the DSC peaks can be achieved; during each heating–cooling cycle, DNA has been incorporated in the membrane in higher and higher concentrations leading to the establishment of a “uniform” fluid phase without detectable phase transitions.[14] Typical thermograms from MLVs interacting with plasmid DNA are presented in Figure 1, where the previously mentioned thermal behavior is expressed in a lower degree than for LUVs because of multilamellarity. Even so, the complexity of the system and the interactions bring to surface the weaknesses of the DSC technique and point out the need for complementary techniques.

Very important for understanding biological functions in the cell nuclei as well as for biotechnology applications are the interactions of RNA with lipid bilayers. A very systematic study was recently published aiming at understanding how tRNA (baker’s yeast, 60-90 bp) associates with DMPC membranes doped with the anionic lipid 1,2-dimyristoyl-sn-glycero-3-phospho-l-serine (DMPS) or the cationic amphiphile DODAB. The work was carried out by Michanek *et al*. by combining DSC with a quartz crystal microbalance with dissipation (QCM-D) techniques.[45] Poly-A short single-stranded DNA (63 bp) and double-stranded salmon sperm DNA (2000±500 bp) were also assessed for adsorption on lipid bilayers for comparison. tRNA was found to adsorb on DMPC liposomes after long incubation times with maximum adsorption taking place when incubation is performed at the liquid-crystalline (*L_α_*) phase of the membrane as also observed by Koiv *et al*.[46] Divalent cations did not affect tRNA adsorption. Adsorbed tRNA insignificantly increased the main-phase transition temperature implying that neither hydrophobic nor strong electrostatic interaction was taking place, although an increase in cooperativity was observed, as ∆*T*_½_, that is, the transition peak temperature width at half ∆*C*_p_ maximum, decreased from 1.6°C to 1.3°C. This weak binding was attributed to apolar interactions between tRNA and the apolar parts of the lipid exposed at the hydrophilic zone of the bilayer.

Miscibility of DMPS was also assessed with DSC, giving a single and strongly cooperative peak for all DMPC-DMPS mixtures implying narrow coexistence regions for the two phases L_β_ (gel tilted) and L_α_. The presence of tRNA in DMPS mixtures, at a monovalent Na^+^ buffer, increased *T*_m_ significantly, broadened the transition peak, and in some cases caused the splitting of the peak. The intensity of these effects increased with the relative DMPS content in the mixture and the tRNA concentration. The broadening and the splitting of the transition peak were explained by lipid segregation. In this case, the repulsion is driving this segregation and not the attractive forces as studied in other works presented. DMPS lipids are repelled by the nucleic acid negative charges and they are segregated from the DMPC lipids, leading to the formation of DMPC-rich domains exhibiting lower transition temperature than the ideally mixed DMPC-DMPS. Those effects were maximum and permanent after the third heating cycle, implying that the system tRNA–liposomes was not at a steady state immediately after mixing and incubating at a stable temperature, but it required the lipid bilayers to undergo the transition from order to disorder twice for a stable state to be established. This supported the hypothesis that the exposed apolar parts of the bilayer are interacting with apolar moieties of tRNA.

In contrast to these results, DODAB (5%) doped DMPC membranes reached a stable state in only minutes after mixing with nucleic acids due to the strong interaction between the cationic compound and the negative charges of the nucleic acids. The effect of tRNA on the main-phase transition of DMPC-DODAB was analogous to the DMPC-DMPS membranes, but driven by electrostatic attraction. The main characteristic of the DSC thermograms is the peak splitting which indicated the tRNA mediated segregation and the formation of DODAB-rich domains; DODAB domains associated to tRNA were also confirmed with solid NMR experiments.[47] DODAB-rich and DMPC-rich domains were characterized by different temperatures shifted towards the pure DODAB (36.0°C and 44.0°C) or DMPC (24.0°C) melting temperatures, respectively.[48] A point of difference between the two segregation cases – weak repulsive or strong attractive forces – was the concentration of the tRNA which was 10 times lower for the cationic membranes but it was enough to cause drastic changes to the lipid bilayer.

When assessing different nucleic acid interactions with DMPS containing membranes, similar effects have been observed only for the single-stranded DNA of comparable size to the tRNA (ssDNA_63_) most probably because it is more agile and it contains unpaired bases as tRNA. Larger DNA molecules, either double-stranded or single-stranded, left the main-phase transition of DMPC or DMPC-DMPS binary liposomes unaffected. Conclusively, it has been shown by DSC that tRNA induces lipid segregation either by attractive or repulsive forces. Details from the nucleic acids–membranes interaction as induction of disorder in the lipid bilayer or lipid headgroup interconnection and segregation can be inferred by DSC experiments. Nevertheless, a second experimental technique as nuclear magnetic resonance (NMR) or small-angle x-ray scattering (SAXS) must be employed in order to verify the proposed interaction model.[47]

One of the earliest systematic studies of DNA and RNA effects on thermal phase behavior of multilamellar and unilamellar liposomes, employing DSC, was conducted by Koiv *et al*.[46] Liposomes composed of the zwitterionic lipid DMPC doped with the cationic single-tail lipid sphingosine at various ratios where studied for calf thymus DNA and RNA effects. Vesicles containing neat DMPC showed again no effect on both pretransition (gel to ripple phase – *L*_β_ → *P*_β_) and main-phase transition (ripple to liquid-crystalline phase - *P*_β_ → *L*_α_) by the presence of DNA and RNA. Introducing sphingosine in the PC bilayers resulted in nucleic acid binding and significant changes in the thermal profiles. Phase separation was observed by the splitting of the main-phase transition peak. Lipid segregation was manifested by shifting the phase transitions toward higher temperatures implying the formation of sphingosine-rich domains. At the same time, the reduction of the total enthalpy at increasing DNA concentrations and the abolishment of the initial – lower temperature – phase transition peak indicates DNA-imposed disorder in the lipid bilayer.

In connection to the previous studies of PC-containing membranes, calorimetry was combined with small-angle neutron scattering (SANS) and SAXD to study the interaction of 1,2-dipalmitoyl-sn-glycero-3-phospho-l-choline (DPPC) liposomes, mediated by Zn^2+^, with salmon sperm fragmented DNA (0.5–1 kbp) by Uhrikova *et al*.[30] Structural information from the DPPC-DNA-Zn ^2+^ complex was correlated to the thermotropic phase transitions in relation to Zn^2+^ concentration. Zinc cations bind strongly to PC and induce dehydration as well as conformational changes to the hydrophilic part of the DPPC lipid bilayers.[49,50] At the same time, zinc can condense DNA, like other divalent cations, and thus it can bridge PC headgroups with DNA polyanions.[51] The DPPC molecular order was almost unaffected by the presence of DNA in a monovalent buffer environment as indicated by the *T*_m_ of the pretransition and main-phase transition, in contrast to Suleymanoglu 2004 results where DNA had severe impact on DPPC bilayers.[52] At low zinc concentrations, the DSC trace of the DPPC-Zn^2+^-DNA complex was altered substantially. The pretransition peak was reduced to the detection limit and the main-phase transition had a slight temperature increase, and it appeared as a shoulder over a new high-enthalpy peak at higher temperature. The high-temperature peak was assigned to the phase transition of the DPPC-Zn ^2+^ -DNA complex, and the lower transition was the main-phase transition of DPPC-Zn ^2+^. These assignments were also inferred by SANS and SAXD experiments. Two lamellar phases were confirmed; the first was attributed to the DPPC-Zn^2+^ lamellar structure, and the second to the DPPC-DNA structure in which DNA strands are intercalated in the aqueous planar space between sequential DPPC bilayers. The enthalpy content for each transition could be deconvoluted to quantify the two phases. That analysis showed that at low zinc concentration, as of 1 mM, there was maximum DNA complexation. Further increase in zinc cations to 20 mM Zn^+2^ significantly decreased the amount of complexed DNA, as DNA was neutralized by the excess zinc cations. At high zinc content, a low-enthalpy peak manifested the presence of DPPC-DNA complex. DSC data analysis also defined the zinc–DPPC interaction saturation point and stoichiometry to three cations per DPPC molecule.

In another recent study by Xu and Anchordoquy, DNA carriers composed of DOTAP and high cholesterol content exhibited interestingly high gene delivery efficiency.[53] DSC was employed to investigate the anhydrous crystalline phase of cholesterol formed over 60% and the effects of bound DNA.[54] The anhydrous neat cholesterol domains or “crystallites” exhibit a phase transition of around 40°C, which were not affected by DOTAP-bound DNA. Moreover the cholesterol domains transition enthalpy change was unaffected at high cholesterol content. Even though, an enthalpy decrease trend with high uncertainty was observed for increasing DNA amount in cholesterol 70% vesicles; this could be possibly due to DNA-induced disorder at the cholesterol domains interface.

DMTAP-DMPC binary multilamellar liposomes and their complexes with calf thymus DNA have been thoroughly studied utilizing DSC in combination with SAXS and WAXS by Zantl *et al*.[31] The phase behavior of the binary lipid mixture at various molar ratios was explored with DSC as well as their complexes with DNA, and detailed phase diagrams have been created. The phases observed were lamellar as confirmed by the structural experiments. The basic DNA effect was the increase in transition temperatures as DNA stabilized the membranes at the isoelectric DNA to cationic lipid ratio. Additionally, at low TAP content, lipid demixing was reflected as a phase transition peak splitting toward higher temperatures compared to lipid mixtures in the absence of DNA. The applicability value of the phase diagrams is realized in the following study by Koynova *et al*., where binary lipids phase diagrams were correlated to transfection efficacies.

Extensive DSC studies of numerous binary lipid formulations with potential transfection applications –ethylphosphatidylcholines, dimethylammonium bromides, DMTAP, egg PC, 1,2-dioleoyl-sn-phosphatidylglycerol (DOPG) – was conducted by Koynova *et al*.[55,56] Phase diagrams of binary lipid membranes – self-organized in lamellar phases – were correlated to transfection efficiencies and revealed that maximum delivery efficiency was for binary lipid formulations that exhibit gel-liquid crystalline phase coexistence at physiological temperature. In line with the latter were the results of novel synthetic cationic amphiphiles applied for the *in vitro* lipofection of mouse melanoma cell lines.[34]

It must be mentioned at this point that DSC has been employed in several cases to characterize the thermal denaturation of DNA in relation to membrane binding, where increasing melting temperature indicated enhanced stability for the formed lipoplex.[46,57–59] Lobo *et al*. have showed that DNA was thermally stabilized by DAB cationic lipids more than TAP lipids showing a shift from 109 °C to 122 °C.[58] This effect was bidirectional in terms of DNA-induced lipid order; DNA increased the main-phase transition temperature of DDAB by 4.8 °C, while the equivalent increase for 1,2-distearyl-trimethylammonium propane (DSTAP) was only 2.0 °C. Additionally, they have found that DOPE abolished the stabilization in contrast to cholesterol which had no effect. Finally, in another study, DSC has also been employed to investigate hydration levels of cationic lipids–DNA complexes in the absence and presence of helper lipids as a complementary technique to laurdan fluorescence generalized polarization by Hirsch-Lerner and Barenholtz.[37,60,61] Cationic lipids used in that study were DOTAP, DMTAP, and DC-Chol (3β-{*N*-[(*N*´,*N*´-dimethylamino)ethane]-carbamoyl}cholesterol) while for helper lipids DOPE, DOPC, or cholesterol where chosen.

## Correlation of DSC Data with Other Techniques

DSC can provide information regarding thermally induced phase transitions and with that as a probe information about DNA and membranes interactions can be extracted. DSC cannot give direct lipid–DNA structural information and must be combined with other experimental techniques in order to interpret the acquired calorimetric data with greater confidence.

As it was mentioned earlier, in the study by Koynova *et al*., lipid phases were assessed relatively to their efficiency for DNA delivery.[55] In yet another extensive study though, DNA delivery efficiency could be correlated to lipid–DNA complex phases.[62] Structural information of the lipid phases was extracted by SAXS. Mixtures of the cationic lipids ethyldilauroyl-O-phosphatidylcholine-ethyldioleoyl-*O*-phosphatidylcholine (EDLPC-EDOPC), DOTAP-DOPE, and their lipoplexes were assessed. Anionic lipids – phosphatidylglycerol, phosphatidylserine, and cardiolipin – were mixed and characterized in connection with DNA dissociation and delivery. The molecular structure of the lipids and the consequential apolar-polar interface curvature regulated the final structural conformation. Lipoplexes of EDLPC-EDOPC exhibited lamellar conformation; EDLPC-EDOPC-cardiolipin exhibited inverted micellar cubic – the highest interfacial curvature – while the less-effective mixtures EDLPC-EDOPC-cardiolipin exhibited inverted hexagonal, cubic bilayered, and/or coexisting lamellar phases. The most widely studied mixture, DOTAP-DOPE, at low temperature appears to be, while at higher temperatures exhibits an inverted hexagonal phase. Early SAXS structural studies by Lasic *et al*. on DODAB-cholesterol-DNA complexes revealed a bilayered packing with dehydrated DNA ordered in a 2D layer between the cationic lipid bilayers.[63]

Very few studies employ only DSC for the investigation of membrane–DNA interactions.[52] Most of the studies combine SAXS, WAXS, SANS, or other structural techniques like cryo-EM and solid-state NMR.[30,31,47,63,64] In some other cases, binding assays have been conducted employing QCM-D or ITC in order to add pieces to the lipids–DNA interaction puzzle.[43,46,65]

## Summary

The power of DSC as an experimental tool for characterizing nucleic acids–lipid membrane interactions is projected throughout all the studies reviewed here. DSC peaks associated with phase transitions or with the thermal denaturation of nucleic acids are used as the principal experimental evidence in all the studies. Straightforward analysis provides information on the enthalpy, the transition temperatures, and the cooperativity of the lipoplex phases. Most of the studies conducted involved binary lipid mixtures. It is essential, as a first step, to study the miscibility of the two lipids by DSC and characterize the phase transitions for safe interpretation of the experimental data from the DNA or RNA impact. In most cases and at low DNA concentration, surface-bound DNA will stabilize the lipid membranes through electrostatic interactions with the polar headgroup, though always depending on the membrane fluidity. When DNA penetrates the lipid membrane, the results are severe; the interactions can be slow and the fluidity of the lipid phase is almost always enhanced whilst lipid interdigitation is most surely abolished as is the lipid pretransition. In the binary lipid mixtures, one of the two components is the DNA-binding partner, while the other plays either a stabilizing or a destabilizing role for the long-range molecular organization. Thus, DNA is anticipated to demix lipids. Indeed, lipid demixing and segregation take place in most charged-zwitterionic binary systems resulting in phase-separated domains. Another issue accentuated is the concentration effect of nucleic acids; at low concentration, DNA can induce long-range order, while at high concentration it induces disorder. Information about the kinetics and the nature of the nucleic acids–membranes interactions can also be extracted with DSC. For very weak or slow-kinetics interactions, the establishment of a stable state can be monitored and demonstrated by successive heating and cooling scans and the repeatability of the so-obtained DSC thermograms. In several studies, the steady state is established after long incubation times and a large number of heating and cooling cycles. Since nucleic acid–membrane interactions also involve apolar interactions, several passes from order to disorder of the lipid bilayer will enhance apolar–apolar interactions and assist the establishment of a new phase for the complex. Recent developments in gene delivery involve novel cationic delivery reagents as amidines, polyethylenimines (PEI), and commercially available reagents (Polyplus-transfection SA, 67401 Illkirch Cedex, France) as well and exhibit increased transfection efficiencies.[66–68] An extensive biophysical approach on these novel gene carriers can also contribute to a better understanding of the mechanisms behind improved transfection.[69,70]

DSC is a valuable technique for studying DNA–lipid complexes, which exhibits many advantages such as the relatively easy-to-use experimental setups and the straightforward data analysis. As a technique, DSC is efficient and relatively of low cost, reliable as an ensemble technique, and most importantly it can assess systems in physiological conditions. DSC should be used in combination with other techniques like structure analysis techniques or binding assays. Once the thermal behavior of a system is correlated with structure facts, then DSC can be used independently. Both qualitative and quantitative analysis can be carried out with DSC, contributing to the elucidation of biological mechanisms as well as to the development of improved gene carriers.
